# Elbow Joint Hybrid Assistive Limb Treatment Improves Upper Limb Dysfunction in Guillain-Barré Syndrome: A Case Report

**DOI:** 10.7759/cureus.68586

**Published:** 2024-09-03

**Authors:** Koji Hayashi, Takumi Matsuyama, Mamiko Sato, Asuka Suzuki, Yuka Nakaya, Naoko Takaku, Toyoaki Miura, Yasutaka Kobayashi

**Affiliations:** 1 Department of Rehabilitation Medicine, Fukui General Hospital, Fukui, JPN; 2 Graduate School of Health Science, Fukui Health Science University, Fukui, JPN

**Keywords:** robot-assisted therapy, robot-assisted rehabilitation, robot assistive training, rehabilitation training, guillain-barre syndrome (gbs)

## Abstract

Robot-assisted rehabilitation is becoming an important option in rehabilitation medicine. We utilized one such device, the elbow joint hybrid assistive limb (HAL), for a patient with Guillain-Barré syndrome (GBS). The patient was a 64-year-old man, 16 months post-onset of GBS. Due to severe neuropathy, he was completely dependent on others and unable to eat independently. After approximately two weeks of intensive rehabilitation and self-training following his hospital discharge, he regained the ability to feed himself. This report highlights the effectiveness of HAL in the chronic phase of GBS.

## Introduction

Guillain-Barré syndrome (GBS) is an inflammatory disease of the peripheral nerves that can lead to acute flaccid paralysis, primarily affecting the limbs [1,2]. An annual global incidence of GBS is estimated as approximately one to two per 100,000 person-years [1,2]. The main cause of GBS is an abnormal immune response following infection such as viral or bacterial infection, particularly Campylobacter jejuni that causes peripheral nerve involvement [2]. The clinical symptoms of GBS include rapidly progressive bilateral weakness of the legs and/or arms, areflexia, and distal paraesthesias or sensory loss [1]. Disease onset is acute or subacute, and patients typically reach maximum disability within two weeks [3]. GBS is treated with immunotherapy such as intravenous immunoglobulin or plasma exchange therapies and rehabilitation therapy.

In the field of neuromuscular disease rehabilitation, the effectiveness of robot-assisted rehabilitation has been reported for a variety of conditions, including Parkinson’s disease, stroke, cavernous malformation, hypertrophic olivary degeneration, severe acquired brain injury, and spinal cord injury [4]. As far as we know, there is only a report of robot-assisted rehabilitation for GBS, and its endpoint of improving is walking ability [5]. One of the robot-assisted devices, the hybrid assistive limb (HAL), is a rehabilitation device that operates on the principle of "interactive biofeedback," enabling it to synchronize its movements with the user's intended motion and the sensory input generated by HAL-assisted actions [6]. In this report, we describe a case with GBS treated with elbow joint HAL to relieve upper limb dysfunction.

## Case presentation

A 64-year-old man, who had a history of diabetes mellitus, hypertension, and glaucoma, developed diarrhea following eating raw beef liver. Ten days after this episode, he suffered from numbness and muscle weakness in both lower limbs and was admitted first hospital. On day two, he was not able to gait for weakness. On day three, he developed respiration failure and disturbance of consciousness (Japan Coma Scale: III-300) and was transported to the Department of Neurology in the second hospital. He was diagnosed with GBS due to progressive muscle weakness (distal dominant), areflexia, and respiratory failure. Although the result was proved several days later, anti-GM1 and anti-GQ1b antibodies were detected in his blood serum. Although he was treated with seven times simple plasma exchanges under intubation and a respirator, laryngopharyngeal, respiratory muscle, and severe limb/trunk muscle paralysis remained. He was transported to our hospital for rehabilitation on day 80 after admission. Vital signs showed clear consciousness, body temperature of 36.9 degrees Celsius, heart rate of 81 per minute, blood pressure of 123/69 mmHg, and saturation of percutaneous oxygen of 95% under respirator after tracheostomy. Neurological examination on the admission to our hospital revealed communication failure (he can use only head-shake gestures), bilateral facial nerve and laryngopharyngeal palsy, the Medical Research Council (MRC) grade 2 muscle weakness in the proximal muscle (trapezius, sternocleidomastoid, neck flexor/extensor, four extremities), grade 3 muscle weakness in the distal muscle (four extremities), and severely limited range of motion (ROM) and areflexia. Neither sensory disturbance nor autonomic dysfunction was noted. Hughes's grade used to assess functional disability was grade 5. The Modified Erasmus GBS Outcome Score (mEGOS) was assessed as nine points. We started rehabilitation therapy, including physical, occupational, and speech therapy for a total of 180 minutes per day. He was able to be weaned off the ventilator and start feeding training one month and two months, respectively, after admission to our hospital. He enthusiastically engaged in rehabilitation, and his activity of daily living (ADL) gradually improved. At discharge from our hospital six months after admission in our hospital, a neurological examination revealed his muscle strength was improved up to MRC grades 2-3 in the upper limbs and grades 3-4 in the lower limbs. Additionally, his Barthel Index on the ADL scale was improved from zero (on admission) to five (at discharge), and he can maintain a sitting position. Subsequently, he entered a nursing home. Although the rehabilitation environment was not adequate in that facility, he continued to receive rehabilitation treatment (for about 20 minutes per day, three days per week). Eleven months after the initial onset, he was able to eat three meals a day. Sixteen months after onset, he was admitted to our hospital for treatment with an elbow joint HAL. Neurological examination on second admission revealed MRC grades 2-3 in the upper limbs and MRC grades 3-4 in the lower limbs. Regarding his ADL, he was able to propel himself in a wheelchair with hand rims. We planned his rehabilitation treatment with HAL, with the goal of having meals on his own (Table 1). He performed elbow flexion and extension exercises with HAL, including reach exercises up to the mouth and training on using the spoon. In addition to manual joint ROM exercises for the upper limbs and task-oriented training using a portable spring balancer (such as reaching movements towards the mouth, peg manipulation, rolling up a newspaper, and practicing with a spoon), the flexion/extension exercise was started in the bilateral elbows from 200 times a day and gradually increased it up to 500 times a day. As a result of rehabilitation treatment with HAL, ROM of the bilateral elbow joints and forearm supination increased with both passive and active movements (Table 1). In addition, he had difficulty eating on his own on admission, but by the time he was discharged from the hospital, he was able to bring a spoon to his mouth (Video 1). He was discharged from our hospital on day 18. The flexion/extension exercise in the bilateral elbows was continued with HAL at his home 1,000 times a day. He was able to eat on his own 20 days after being discharged from the hospital (Video 1).

**Table 1:** Diagram of the plan and outcomes of rehabilitation therapy using the hybrid assistive limb (HAL) after admission and discharge. After rehabilitation therapy with HAL, there was an increase in the range of motion in the elbow joints and forearm supination and an improvement in feeding movements.

Status	Hospitalization							Home care	
Day	4	6	7	8	11	12-13	14-18	~10	20
Upper Limb MRC grade	2-3 2-3							2-3	
Rehabilitation elbow joint movement		200 repetitions/day right side	300 repetitions/day each side	400 repetitions/day each side		500 repetitions/day each side		1000 repetitions/day each side (Self training)	
Meal	Unable to tolerate oral intake independently				Able to reach mouth with spoon using a spring balancer		Able to reach mouth without using a spring balancer		Able to tolerate oral intake independently
Elbow ROM (L/R)	Active: 100°/100° Passive: 110°/110°						Active: 115°/115° Passive: 120°/130°		
Forearm pronation ROM (L/R)	Active: 90°/90° Passive: 90°/90°						Active: 90°/90° Passive: 90°/90°		
Forearm supination ROM(L/R)	Active: 15°/20° Passive: 15°/30°						Active: 50°/50° Passive: 60°/70°		

**Video 1:** The video records a comparison of the patient's condition before and after treatment with the elbow joint hybrid assistive limb. The patient's elbow range of motion and feeding movements improved remarkably following the treatment.

## Discussion

We presented a case of GBS in which rehabilitation therapy using elbow joint HAL was administered to the bilateral elbow joints. This treatment was initiated approximately one year after the onset of symptoms and was evaluated as effective. Regarding the prognosis of GBS, the majority of cases, even severe cases, show extensive recovery within one year after onset [1]. Approximately 80% of GBS cases recover the ability to walk six months after onset [1]. In spite of the favorable prognosis for the majority of cases with GBS, fatal events occur in 3-10% of GBS cases, primarily due to cardiovascular and respiratory complications [1]. In addition, long-term residual symptoms are often noted, including neuropathic pain, weakness, and fatigue. Notably, the recovery process of these symptoms may occur >5 years after disease onset [1]. The mEGOS is commonly used as a tool to assess the prognosis of walking ability in GBS [7]. In our case, he gained nine points on mEGOS on admission with the age of 60 years, the presence of preceding diarrhea, and an MRC sum score <30, which meant a poor prognosis. In fact, one year after onset, the patient remained severely quadriplegic and was unable to walk or eat on his own, although his symptoms had still mildly improved. As his symptoms improved slightly, he required new rehabilitation treatment to improve his ADL. He began his rehabilitation treatment using HAL by renting it at his own expense, at his own request.

Upper limb type of HAL has been used for various diseases and its efficacy has been reported, including spastic cerebral palsy [8], brachial plexus injury [9], post-stroke [10], post-operative cervical spinal cord paralysis [11], and spastic hemiplegia [12]. The detailed mechanisms for the effectiveness of HAL against neurological disease remain unknown, but one hypothesis is that the powerful assistance provided by HAL makes it easier for muscles and nerves to obtain a motor learning effect [8,9]. HAL's implementation of joint motion assistance, which involves applying assistive joint torque in real-time based on muscle activation, along with adjusting the weight parameters of agonist and antagonist muscles, facilitates the characteristics required for simple and intuitive motion of elbow flexion and extension [8]. As muscles and nerves become successful in achieving enough muscle contraction with HAL, they may eventually become capable of performing sufficient contractions without the assistance of HAL.

To our knowledge, there is no documented evidence of the efficacy of HAL for GBS. Although the effectiveness of the robot suits other than HAL against GBS has been previously published, it is limited to its effectiveness against gait disorders, such as robot-assisted gait training [5,13]. Notably, previous study suggests that robot-assisted training is effective not only for central nervous system diseases but also for peripheral nervous diseases including GBS. It is no surprise that robot-assisted training has been effective for the upper limbs as well in a patient with GBS, given previous reports. Even after one year from the onset, there is still potential for improvement in neuromuscular function in GBS patients, so proactively introducing robot-assisted training such as HAL may be an option for rehabilitation therapy.

As a limitation, this study involves a single case, and the therapeutic intervention during the recovery process of GBS makes it difficult to distinguish between natural recovery and the effects of the intervention. However, both the patient and we feel that there was a definite and significant improvement in a short period. Before using HAL, neither the patient nor we could imagine the possibility of the patient being able to eat independently. The fact that this was achieved within several days suggests the effectiveness of rehabilitation therapy using HAL. Additionally, while anti-gravity movements were still challenging, the use of HAL made it possible for the patient to engage in sufficient self-training, which is also significant. Moreover, setting a real-life goal, such as regaining the ability to eat, helped increase the patient's motivation. The effectiveness of rehabilitation therapy during the recovery process of peripheral nerve damage caused by GBS has been documented in the past [14]. We believe that, in this case, neurological recovery was also further promoted by using HAL.

## Conclusions

We conducted rehabilitation therapy using the elbow joint HAL on both elbow joints in a patient with GBS one year after the onset of symptoms and confirmed its effectiveness. Robot-assisted rehabilitation therapy is considered effective not only for gait disturbances in patients with GBS but also for upper limb function. In addition, the efficacy of HAL was confirmed in the chronic phase of GBS. To verify the effect of the upper joint HAL on GBS, we need to accumulate more cases.
